# Annotations

**Published:** 1898-07-02

**Authors:** 


### ANNOTATIONS.

### The Hospital Bazaar.

WE heartily congratulate Mrs. Spender, the parent of the Press Bazaar, upon a genuine success. There is good reason to hope, when the accounts are closed, that the London Hospital will benefit to the extent of £10,000. Everybody has worked with a will, and all are entitled to the grateful thanks of everybody interested in the hospital. Mrs. Spender's bright idea, carried out as it has been with such splendid energy and unsparing labour on her part, leads us to ask why not a Press Bazaar once every five years for all the hospitals? Then no great paper would hold aloof from the enterprise, if the reason for abstention on the present occasion be, as the *Times* hints, that such united action on behalf of one hospital was undesirable if not impossible. The Princess of Wales has very graciously aided the present bazaar, and the Prince of Wales's Hospital Fund makes grants to all reputable hospitals, and would so enable a united effort by the Press once in five years to utilise the money raised to the best advantage in the cause of the sick. We hope, therefore, that this suggestion will commend itself to everybody, and that before the committee of the Press Bazaar dissolves steps may be taken to gather up the experience gained for future use, should a united Press Bazaar on behalf of the Prince of Wales's Fund take practical shape next year. If time were taken to organise such a movement at the Imperial Institute, or some other suitable building, it should yield every five years at least £20,000, or, say, £4,000 a year, to the metropolitan hospitals. Mrs. Spender, in any case, is to be warmly congratulated, whether her idea takes permanent root or not.

### A Whiff of Tobacco.

WE do not want to rail at tobacco, but just to draw a little moral from a whiff of smoke. Go where one will the nose, the eyes, and even the throat and bronchial tubes are assailed by smoke, that is by a cloud of particles, every one of which has come direct out of someone else's mouth. It is not a nice idea, but let us see what else it teaches. The fumes do but make visible what is happening all the day whether we smoke or not. Each of the tiny particles of carbon or condensed vapour which in their millions make up a wreath of smoke, does but indicate the track taken by a corresponding particle of expired air, which, if it can carry the visible carbon, can still more easily carry the invisible microbe. Thus a whiff of smoke entering our nostrils and penetrating our lungs does but show the course which might be taken just as easily by a swarm of microbes, and serves to demonstrate one at least of the ways in which a crowded life passed in close community with our fellows leads to mischief. The passage of a whiff of smoke from mouth to mouth does, in fact, but illustrate the mode in which the well-recognised evils of rebreathing expired air are produced. It is not the air but what the air carries with it that does the harm. What is illustrated by tobacco smoke is sometimes proved in another way. In the bright sunbeams motes are said to dance, and by careful watching one may see not only how numerous these motes are, but of what nasty stuff they are not infrequently composed. The wheezy flower seller coughing over his tray of violets, the loud voiced hawker shouting over his barrow of strawberries, the sniffling child sneezing at the street corner, the panting person who *will* shake out his handkerchief in the 'bus before using it, even polite people talking to each other, are all doing things which on a dull day seem innocuous enough. Let the sun shine, however, and the tell-tale sunbeams soon display the showers of saliva and the crowds of dust which are thus scattered in the air and can almost be traced from mouth to mouth. This is æsthetically abominable, but in the vast majority of cases probably does no harm. Here and there, however, these particles come from people who are diseased, and carry diseases to those who are healthy. The rebreathing of expired air is certainly one cause of disease, especially to those who live in towns and in close dwellings; and how real is the risk, and how readily the passage of solid particles from man to man and from mouth to mouth is accomplished, is made manifest every time a whiff of tobacco makes us cough.

### The Conscientious Objector.

THE proceedings in the Grand Committee of the Vaccination Bill contain a little bit of evidence as to the working of a "conscience clause" which is of considerable interest at the present time, when so much effort is being made to introduce amendments into the Bill, having for their object the emancipation of the conscientious objector from all compulsion in regard to vaccination. Mr. Chaplin said it happened that at the present moment the Local Government Board had some experience of the working of such a clause, for the guardians of Barton Regis, pending fresh legislation, had taken the law into their own hands, and had issued a circular to those persons whose children had not been vaccinated in which it was said that the guardians would favourably entertain the objections of those who furnished on a proper form a declaration signed and witnessed before a magistrate as to their conscientious objection to the operation. That was to say that the guardians would take no further steps in the matter. Now, what was the effect of this action? First, there was an increasing demand for these forms, with the result that parents who had been in the habit of getting their children vaccinated as a matter of course and without any mishap, now applied for the forms, and had their children vaccinated no longer. Conscientious objectors were said to be springing up like mushrooms, and whole districts were in danger of remaining unvaccinated altogether. To show how rapid had been the growth and spread of this officially authorised system of non-compliance with the law, it was mentioned that the system only began in 1896, and out of 300 cases reported in default in the last two quarters of 1897, no less than 183 had availed themselves of this expedient. Under such circumstances, Mr. Chaplin very fairly asked, if this was what was being done while the system was illegal, what was likely to be the case if such a plan were sanctioned and embodied in the law? At the present time it appears to be the case that one-third of the total number of children born remain unvaccinated, and it is not difficult to see that if a mere statutory declaration of conscientious objection is to set people free from all obligation in the matter, proper preventive vaccination will be at the mercy of any association which chooses to oppose its being carried into effect, and that the vaccination of the future will consist mostly of panic vaccination in the face of epidemics—a most evil and futile consummation to all the efforts that have been made to rid the country of so serious a pest as that of small-pox.

